# Study on unconfined compressive strength and deformation characteristics of chlorine saline soil

**DOI:** 10.1038/s41598-023-50441-5

**Published:** 2024-01-17

**Authors:** Hongru Li, Min Yang

**Affiliations:** 1 Institute of Geotechnical Engineering, Xi’an University of Technology, Xi’an, China; 2 Loess Soil Mechanics and Engineering Key Laboratory of Shaanxi Province, Xian, China; 3 School of Civil Engineering and Architecture, Shaanxi University of Technology, Hanzhong, China

**Keywords:** Engineering, Civil engineering, Microscopy

## Abstract

The distribution of saline soil is wide and the area is large in China. The saline soil in different regions shows their own characteristics. The saline soil of single salt is configured by adding different contents of NaCl to the loess in Xi'an. The effects of chlorine content and water content on soil strength and volume change in a large water content range was studied, and the change mechanism was analyzed by scanning electron microscopy. The results show that the strength of chloride saline soil increases with the decrease of water content. The moisture content of 12% is the critical point of strength change of chloride saline soil. When the moisture content is greater than 12%, the strength of soil decreases with the increase of salt content. When the water content is less than 12%, the strength of saline soil is 3% NaCl content > 1% NaCl content > 5% NaCl content > 0% NaCl content. The volume change of the sample consists of three parts: elastic deformation during sample pressing, volume shrinkage during water loss and salt expansion. In the absence of NaCl and 1% NaCl content, the sample was in the state of shrinkage during the loading process, in which with the decrease of moisture content shrinkage rate slowed down. The volume change rate of 3% and 5% NaCl showed an inflection point from negative to positive when the water content was 15%. When the water content is less than 12%, the saline soil with 3% NaCl content has the characteristics of high strength and unobvious salt expansion. The method of adding 3% NaCl to loess in Xi 'an area can be used to simulate the cement between soil particles, which provides a reference for artificially preparing structural soil. According to the microstructure analysis, the higher the salt content of saline soil, the smaller the soil pores, and the contact form of soil particles gradually develops from the point contact between soil particles to the salt-wrapped structure.

## Introduction

**Figure 1:**
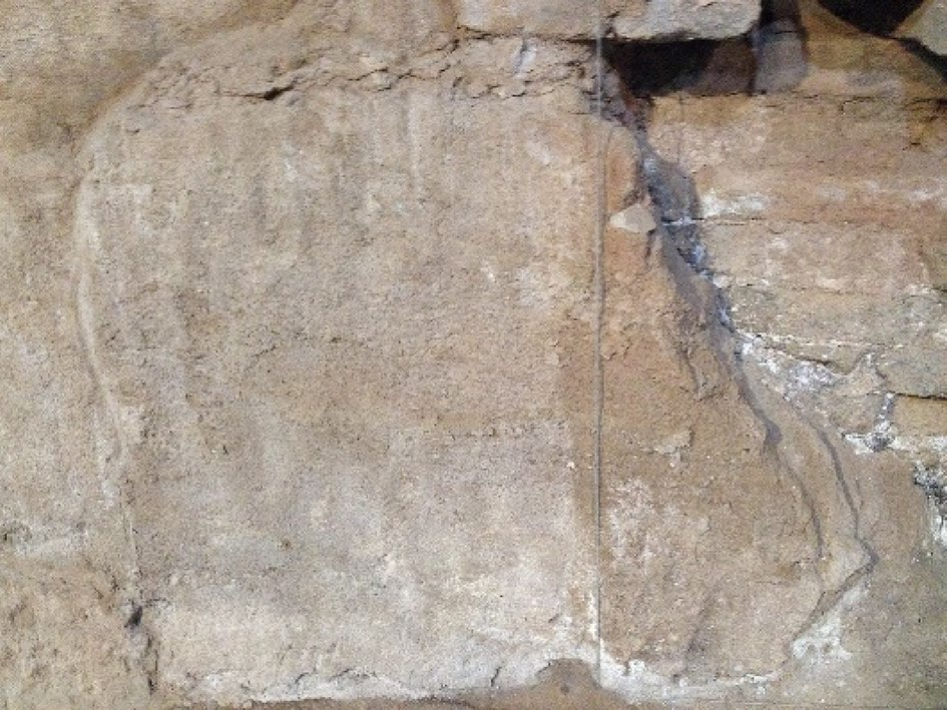
Salt precipitation on the surface of the site.

Whether tropical or cold, southeast coastal or northwest inland, saline soil is distributed in China. Saline soil has different characteristics in different climatic zones. The total area of saline soil is about 36 million hectares, accounting for 4.88% of the available land area in China^1^. When the soluble salt content of loess is more than 0.3%, the collapsible coefficient of soil is large under water immersion condition, which shows strong collapsibility. The loess with salt content greater than this soluble salt content is defined as saline loess^2^. In recent years, with the rise of the groundwater level in Xi'an, the rise of storage level of the moat outside the city wall and the construction of urban hydrophilic environment, a large number of saline disasters have occurred in the ruins of the Tang and Ming emperors' city walls. The salt of the earthen site is released and adhered to its surface, which causes the surface of the cultural relics to fall into pieces of crisp powder and seriously affects the life of the cultural relics, as shown in Fig. 1.

Many scholars have made a series of achievements in the formation of saline soil^3–5^, the water and salt migration laws^5,6^, soil–water characteristic curve^7^, the influence of freeze–thaw cycles on strength and microstructure^8–10^, the relationship between freezing temperature and volume change of saline soil^11,12^, and the influence of stress state on deformation and surrounding structure of saline soil^13–15^, salt content detection and difference of microstructure caused by phase change^16–19^, which provides a theoretical basis for explaining the mechanical phenomenon and disease mechanism of saline soil.

The strength and deformation of soil are the most concerned issues in construction projects^20–22^. As a special soil, the strength and deformation of saline soil are greatly affected by water content and salt content^23^. In order to have a comprehensive understanding of saline soil, it is necessary to carry out the physical and mechanical tests of different salts and composite salts of different proportions under different salt contents in a large water content range. Yu et al.^24^ studied the variation of strength parameters with moisture content of soda-saline soil in the process of water loss and moistening by air-drying and drip method. Liang et al.^25^ analyzed the variation mechanism of soil strength, liquid and plastic limit and difference of surface potentials with chloride content from the microscopic, and explored the strength of saline soil from different aspects, which provided a new idea for subsequent research. As another characteristic of saline soil, salt expansion has also achieved some research results. Most of them are obtained through laboratory tests. Aiming at the sulfate or composite salt containing sulfate with obvious salt expansion effect, a salt expansion calculation formula considering various factors such as salt content, water content, dry density and overlying load is proposed and the parameter sensitivity analysis is carried out. The correctness of the formula is verified by the test results^26^.

In these researches on the strength of saline soil and formulas for calculating salt expansion, only the effect of the decrease of water content on the strength and the salt expansion caused by salt crystallization caused by water loss were considered. These formulas do not consider the inhibitory effect of the dehydrated shrinkage of the sample on salt expansion. This is mainly the research results of saline soil of sulfate or compound salt in a small water content range. These results do not fully consider the differences in soil properties by the influence of water content in the larger interval and the different salts, which affect the strength and deformation of saline soil. In view of the complexity of the composition of natural saline soil, it is difficult to distinguish the influence of different salts on the properties of soil. In this paper, a single salt saline soil was prepared by adding different contents of NaCl to the loess in Xi'an. The influence of chloride content and water content on soil strength and volume change of samples considering water loss shrinkage were studied in large water content range. The reasons for the macro change were analyzed from the microscopic aspect.

## Sample design

### Soil sample preparation

The soil specimens are the saline soil prepared by adding part of NaCl to the remolded loess in the eastern suburbs of Xi'an. The content of each ion in the soil is determined by the method mentioned in the *Specification of Soil Test*. The results are shown in Table 1.

**Table 1:** The ion content in natural loess.

Types	Na^+^	Mg^2+^	Ga^2+^	Cl^−^	SO_4_^2−^	CO_3_^2−^
content/%	0.21	0.15	0.08	0.27	0.11	0.16

The specific making methods of saline soil are as follows: (1) When the water content of the sample is 21%, prepare the water it needs. (2) The saline soil in the test is to integrate NaCl into the water according to three mass ratios of NaCl to soil is 1%, 3% and 5% respectively, and stir it fully. (3) When NaCl was completely dissolved in water, the NaCl solution was mixed in the soil which was dried in advance and passed through a 2 mm sieve. In the process of soil mixing, it is found that the higher the chloride content is, the more likely the agglomerates are to appear in the soil. In order to ensure the accurate and uniform salt content of the prepared soil, it is necessary to pass 2 mm sieve on the prepared soil again. For larger agglomerates, they should be crushed and continue to be sieved until all the soil is sieved.

The prepared soil was sealed in a preservative bag and placed in a moisturizing cylinder for 24 h to ensure that the water and salt were evenly distributed to meet the test requirement. Measurement of moisture content before sample preparation, the wet soil mass is calculated according to the dry density and the moisture content of the test. The cylindrical specimens with a diameter of 3.91 cm and a height of 8.0 cm are prepared by stratification into 5 layers.

### Test scheme

Unconfined compressive stress is the case where the surrounding pressure is zero under triaxial stress. In this paper, SJ-1A strain triaxial apparatus is used to apply axial pressure to the sample without applying peripheral pressure. After many attempts, the shear rate is finally selected as 0.166 mm/min, and the sample is compressed by 0.1 mm to read the measuring force ring once.

The method of making samples with different water content is to spray humidification or air drying of unsaturated samples to test moisture content. The fresh-keeping film is wrapped and sealed with transparent tape to prevent the water loss of the samples or the moisture absorption of the saline soil samples under low water content. The wrapped samples are placed in the moisturizing cylinder for 24 h to make moisture of the sample to uniformly. The saturated sample is made by vacuum saturation method. The specimens are subjected to unconfined compression test, salt expansion and microstructure scanning test. Through these, the effects of water content and chloride content on strength, volume change and microstructure were analyzed. The test scheme is set as shown in Table 2.

**Table 2:** Test scheme.

Parameters	Values
Dry densities (g/cm^3^)	1.4, 1.45, 1.5
Salt contents (%)	0, 1, 3, 5
Water contents (%)	6, 9, 12, 15, 18, 21, 32.78

## Unconfined compressive strength of chlorine saline loess

### Effect of water content on unconfined compressive strength of chloride saline soil

**Figure 2:**
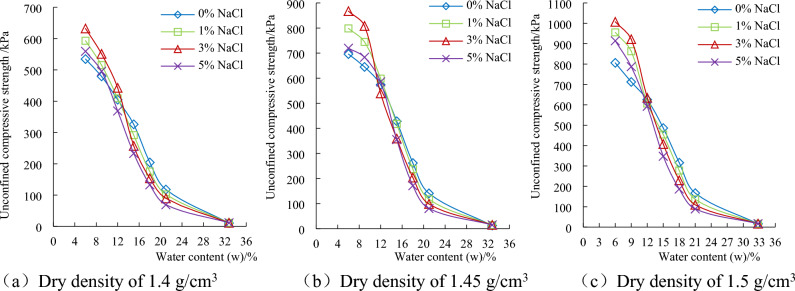
Relationship between soil strength and water content.

The relationship between the unconfined compressive strength (q_u_) of saline soil with different chloride content and water content under three dry densities are shown in Fig. 2. Figure 2 shows that the unconfined compressive strength decreases with the increase of water content in the test water content range. When the moisture content is in the range of 9% and 21%, the strength decreases rapidly, and the strength decreases relatively slowly in the remaining range of water content. Regardless of the dry density and saline soil of samples with chlorine salt content, the moisture content of 12% is the intersection point of its strength change. When the water content is 12%, the strength of soil with the same dry density and different chlorine salt content is basically the same. When the water content is greater than 12%, the higher the salt content, the lower the strength. When the water content is less than 12%, the strength of saline soil with 3% NaCl is the highest, and the strength of soil without NaCl is the lowest. When the water content of the soil sample is 12%, a comparative test of the effect of salt content on liquid and plastic limit and plasticity index was carried out, but the correlation between the two was not found, so it is necessary to continue to carry out in-depth research.

### Effect of NaCl content on unconfined compressive strength of saline soil

**Figure 3:**
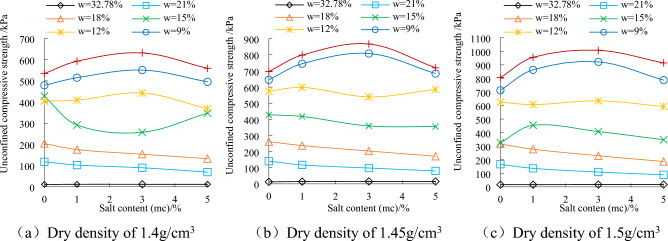
Relationship between soil strength and NaCl content.

Figure 3 shows that the influence of salinity in different moisture content range on unconfined compressive strength is different. At a saturated moisture content of 32.78%, the strength of the soil is low, and the chlorine content has little impact on the strength. When the water content is 21% and 18%, the water content in the soil is higher, and the salinity is still in the dissolved state, which increase the thickness of the hydrated film in the soil and reduce the friction between the soil particles, resulting in the soil strength decreases with the increase of salt content. When the moisture content is 15% and 12%, the soil strength fluctuates slightly with the salinity. This is because some salts begin to crystallize, for the soil with the minor dry density (ρ_d_ = 1.4 g/cm^3^), the cementation between the salt and the soil particles don’t increase the soil strength. When the salinity (5%) is higher, or the dry density (ρ_d_ = 1.45 g/cm^3^ and ρ_d_ = 1.5 g/cm^3^) is larger, salt crystallization enhances the connection between soil particles or fills into pores, which increase the soil strength. The interaction of multi-factor such as salinity, moisture content and dry density makes the soil strength fluctuate when the moisture content is 15% and 12%. When the water content is 9% and 6%, the strength of the soil increases first and then decreases with the increase of the salt content. When the NaCl content reaches 3%, the soil strength is the highest. This phenomenon is caused by the fact that salt crystals enhance the connection between soil particles under low moisture content. Excessive salt crystals (5% NaCl) cause soil expansion and damage the soil structure, which lead to the decrease of soil strength.

The effect of salt content on strength of saline soil with low water content has obvious regularity, while the effect of salt content on strength of saline soil with high water content is irregular. Therefore, the strength problem of saline soil is the result of the coupling effect of water content, salt content and physical and chemical properties. This problem is difficult to explain clearly with a parameter change.

## Volumetric-change characteristics of saline soil

The volumetric-change of saline soil is a complex physical process, which is the result of coupling effect of water, heat, salt and force^27^. The volumetric-change rate δ is the ratio of the difference between the volume after the test and pre-experiment to the sample volume before the test. The calculation method is shown in Formula (1).

1 $$\delta = \frac{\Delta V}{{\mathop V\nolimits_{0} }} = \frac{{\mathop V\nolimits_{1} - \mathop V\nolimits_{0} }}{{\mathop V\nolimits_{0} }} \times 100\%$$

2 $$\mathop V\nolimits_{0} = \pi \left( {\frac{3.91}{2}} \right)^{2} \times 8.0$$

When δ is soil volume change rate, $\mathop V\nolimits_{0}$ is volume of samples, and its calculation formula is formula (2). $\mathop V\nolimits_{1}$ is the volume of the sample after the experiment is completed.

### The influence of water content on the deformation characteristics of chloride saline soil

**Figure 4:**
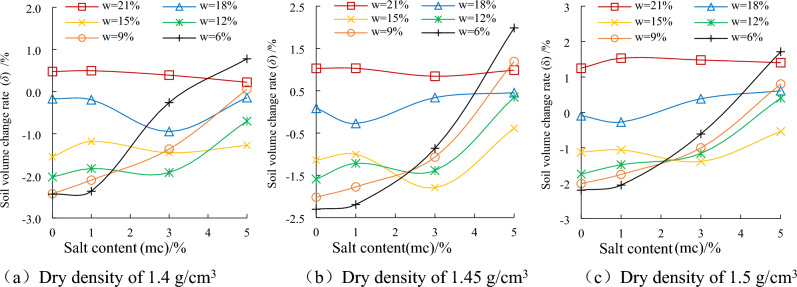
The relationship between soil volume change rate and salt content under different water content condition.

Figure 4 shows that the volumetric-change rate of saline soil is closely related to the salt content of the sample under different water content condition. When the water content of the sample is higher, the salt in the soil exists in the form of solute, which has no effect on the volume change of the sample. When the moisture content of the sample is lower, the body shrinks, which is caused by the volume reduction due to the loss of water. The sample has salt swelling, which is caused by the increase of sample volume due to salt crystallization. The research on saline soil deformation mainly focuses on shrinkage and salt expansion at low water content. For samples with different dry densities, when NaCl is not added and NaCl content is 1%, the volume reduction of the sample shows monotonically increasing with the decrease of water content. This phenomenon is due to the fact that the volume shrinkage of the sample is greater than the salt expansion of the sample at lower salt content, showing a negative volume change rate. When the salt content is 3% and 5%, the effect of salt expansion is more obvious. With the decrease of water content, the volumetric-change rate gradually changes from the initial positive value to the negative value. When the water content decreases to 15%, the volumetric-change rate of the sample is the minimum value. As the water content continues to decrease, the effect of salt expansion is greater than that of the volume shrinkage. The free volumetric-change rate of the 5% NaCl sample gradually recovers from the negative value to the positive value. These test results show that the salt expansion of saline soil is not only related to salt content, but also has a great relationship with water content.

### The influence of NaCl content on the deformation characteristics of chloride saline soil

**Figure 5:**
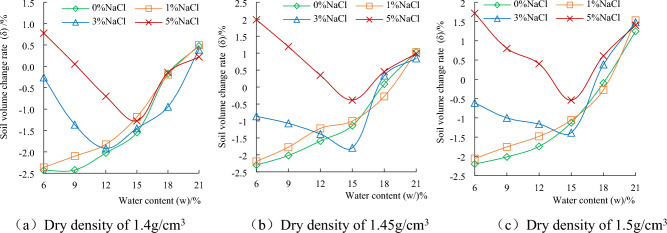
The relationship between soil volume change rate and water content under different salt content condition.

Figure 5 shows that soil volume change rate of samples with different dry densities is positive at 21% water content, and that does not change obviously with the increase of salt content. When the water content is 18%, 15% and 12%, the volumetric-change of the sample fluctuates from volume expansion to volume shrinkage due to the rebound deformation, volume shrinkage caused by water loss and salt expansion of the sample during standing. When the water content gradually decreases to 9% and 6%, salt expansion is the main influence. The volumetric-change rate of the sample changes smoothly from volume shrinkage to volume expansion with the increase of salt content. In the process from volume shrinkage to volume expansion, the higher the salt content and the lower the water content.

According to the analysis of the calculation formula of volume change rate, the volumetric-change rate under the moisture content of the sample should be zero. Figure 4 shows that the volume expansion rate increases with the increase of dry density when the moisture content of the sample is 21%. The reason is that the cyclo-hoop of the sample preparation device during the sample pressing process makes the sample produce elastic compression deformation. During the standing process of the sample, the elastic compression deformation gradually recovered. The greater the dry density of the sample, the greater the rebound deformation.

It is found that for soil samples with high water content, no matter how the salt content changes, the difference of volume change is not obvious. For soil samples with low water content, the difference effect of salt content on volume change is obvious.

## Microstructure analysis

Scanning electron microscope can directly observe the particle shape, location, connection form and pore distribution on the surface of the soil. These microstructures affect the mechanical properties and volumetric-change of saline soil^28–32^. In order to understand the microscopic changes of soil structure after freezing and thawing cycl of saline soil, scanning electron microscopy tests of coarse-grained soil were carried out under the different time of the freeze- thaw cycles, and the microscopic evolution of salt expansion were analyzed through these experiments^33,34^. In order to explain the influence mechanism of NaCl content on soil deformation and strength in this paper, the field emission scanning electron microscopy (JSM-6700F) was selected. The samples with dry density of 1.45 g/cm^3^ and NaCl content of 0%, 1%, 3% and 5% were selected for electron microscope scanning. The samples were cut into cubes of about 5 mm. After the soil samples were sprayed with gold, vacuumed to below 4.4 × 10^–4^ Pa, voltage loaded to 20 kv, focusing and other steps, the microstructure SEM image of the soil samples could be collected. The magnification was 500 times, 1000 times and 2000 times. The representative points can be selected for observation.

**Figure 6:**
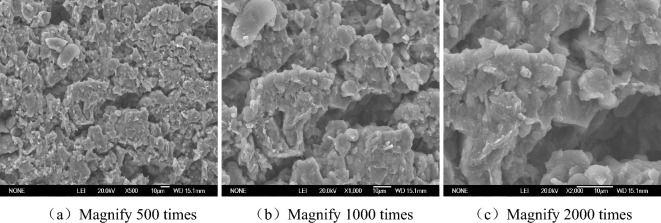
SEM images without NaCl.

**Figure 7:**
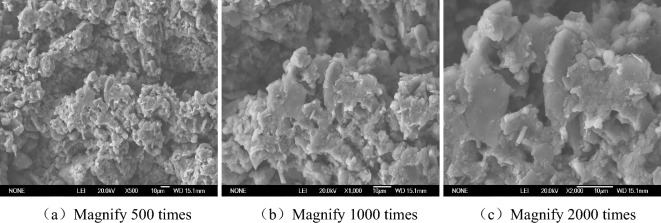
SEM images of 1% NaCl content.

**Figure 8:**
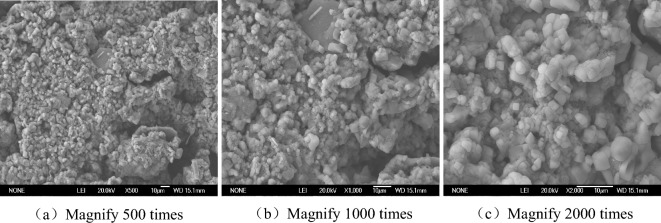
SEM images of 3% NaCl content.

**Figure 9:**
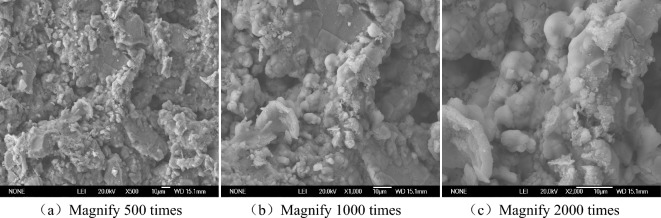
SEM images of 5% NaCl content.

The SEM images of Figs. 6, 7, 8 and 9 can clearly reflect the pore size, and the connection form of crystalline salt and soil particles under different magnification and different salt content. The enlarging the image by 500 times clearly shows the shape of the particle connection and the distribution between the particles and the pores, but the tiny pores and salt crystals are not observed. This picture can only observe the structure of saline soil, but it cannot observe the micro-pores and salt crystals. The observation area of the image magnified by 1000 times is reduced by half compared with the observation area magnified by 500 times. The pore distribution and size can be clearly observed, but the salt crystallization cannot be clearly observed. The image is clear at the magnification of 2000 times. The material showing a smooth surface in the picture is NaCl crystallization. The connection between NaCl and soil particles can be observed. It is found that some NaCl crystals are filled in the pores of the soil.

In the absence of NaCl, the soil pores are numerous and densely distributed, and most of them are connected pore. The soil particles are connected by the original soluble salt crystallization in the soil, which makes the soil strength low and the volume shrinkage obvious. The pores of the soil with 1% NaCl content are smaller than those without NaCl, but the number is comparable. There is only a small amount of salt crystallization on the surface of the soil, which is not obvious for the improvement of soil strength and the inhibition of soil deformation. A large number of cube NaCl crystal can be observed on the surface of soil with 3% NaCl content. Some of these salt crystal fill the original pores of the soil, which reduce the pores and cause the sample to swell. These crystals also act on the bonding of soil particles, enhancing the bonding strength between particles. There are fewer pores on the surface of 5% NaCl soil. In the case of magnification of 2000 times, it is observed that the salt crystals completely encapsulate the soil particles, and a smooth NaCl crystal layer is formed on the surface. The volumetric-change of the soil becomes significantly larger. The measured strength value of 5% NaCl is smaller than that of 3% NaCl and 1% NaCl. Combined with the characteristics of SEM images, the reason why the higher the salt content of soil samples, the lower the strength was analyzed. On the one hand, salt expansion destroys the structure of soil. On the other hand, the soil particles are wrapped by NaCl salt, and the friction strength of soil is reduced, which reduces the strength of the soil. This should be paid attention to in practical engineering.

When the salt content of the soil sample is high, the microstructure of the soil sample is fibreized blocky, there are more fly-over structures, and the solid particle are mainly chloride and other mineral particles of the sheet prismatic. After the salt content of the soil sample is reduced, the microstructure of the soil sample is crumb structure, fly-over structure formed between the solid particles is reduced. The solid particles of the soil sample are refined, there is no flaky salt crystals, and the pores become smaller. It can be seen that the change of salt content will change the pore structure of soil.

## Conclusion

Saline soil is prepared by adding NaCl to loess in Xi 'an. The volumetric-change characteristic and unconfined compressive strength of samples with different dry densities under different salt content and water content condition were studied, and the change mechanism were analyzed from the microcosmic aspect. The conclusions are as follows.

1. Through experiments, it is found that regardless of the dry density and salt content of chlorine saline soil, soil strength increases with the decrease of water content. The moisture content of 12% is the critical point of strength change of chloride saline soil. When the water content is more than 12%, the strength of soil decreases with the increase of salt content, but it is lower than that of the soil without chlorine salt. When the moisture content of the test is less than 12%, the strength of the chloride saline soil is higher than that of the soil without chloride.
2. When the water content is less than 12%, the unconfined compression strength of saline soil under 3% NaCl content is the highest, followed by 1% NaCl, and 5% NaCl is the smallest. Although the addition of chloride salt helps to enhance the strength of soil under low water content, excessive salt content will break the soil structure and reduce the strength of soil.
3. The volumetric-change of chloride saline soil samples is composed of three parts: elastic deformation during compression, volume shrinkage during water loss and salt expansion. In the absence of NaCl and 1% NaCl content, the sample was in the state of shrinkage, in which with the decrease of moisture content shrinkage rate slowed down. The volume change rate of 3% and 5% NaCl showed an inflection point from negative to positive when the water content was 15%.
4. When the water content is less than 12%, the saline soil with 3% NaCl don’t occur bulking, the chloride salt increases the connection between the soil particles and enhances the strength of the soil. If you want to simulate the preparation of artificial structured soil by adding NaCl to simulate the cement between soil particles, it is recommended that NaCl with a mass ratio of 3% should be added to the loess in Xi 'an.
5. The microstructure change of saline soil has a great influence on the strength and deformation of soil. The SEM image show that the higher the salt content of saline soil, the smaller the pore size of the sample, and the contact form of soil particles also gradually develops from the point contact between soil particles to the salt particle wrapping structure. The microstructure changes from the fly-over structure to the crumb structure. The solid particles of the soil sample are refined, there is no flaky salt crystals, and the pores become smaller. It can be seen that the change of salt content will change the pore structure of soil.

## Data Availability

The data used to support the findings of this study are included within the article.
